# Prostate specific membrane antigen PET avidity in a granular cell tumour of the left supraspinatus muscle: a case report

**DOI:** 10.1093/bjrcr/uaae015

**Published:** 2024-05-29

**Authors:** Michael T Hsieh, Farokh Fargah, Abdul Rahim Mohd Tahir, Ngo Tue Le, Thomas P Shakespeare

**Affiliations:** Department of Radiation Oncology, Mid North Coast Cancer Institute, Coffs Harbour, New South Wales 2450, Australia; Anatomical Pathology, Laverty Pathology, North Ryde, NSW 2113, Australia; Department of Radiation Oncology, Mid North Coast Cancer Institute, Coffs Harbour, New South Wales 2450, Australia; Department of Radiation Oncology, Mid North Coast Cancer Institute, Coffs Harbour, New South Wales 2450, Australia; Department of Radiation Oncology, Mid North Coast Cancer Institute, Coffs Harbour, New South Wales 2450, Australia

**Keywords:** granular cell tumour, abrikossoff, granular cell schwannoma, granular cell nerve sheath tumour, granular cell myoblastoma, PSMA, prostate, Positron emission, prostate-specific membrane antigen, PSMA-PET

## Abstract

Granular cell tumour is a rare, mostly benign, soft tissue, neuroectodermal tumour, most commonly seen in the skin and peripheral soft tissue. There are no publications to date of PSMA-PET avidity in a granular cell tumour. In this 60 year old male, staging PSMA-PET for a localized intermediate risk prostate cancer incidentally identified a PSMA-avid left supraspinatus lesion, which was subsequently biopsy-proven as a granular cell tumour. We present the first case of PSMA-avid granular cell tumour and add to the growing literature documenting PSMA-PET avidity in benign and malignant lesions apart from prostate cancer.

## Case presentation

**Figure 1.**
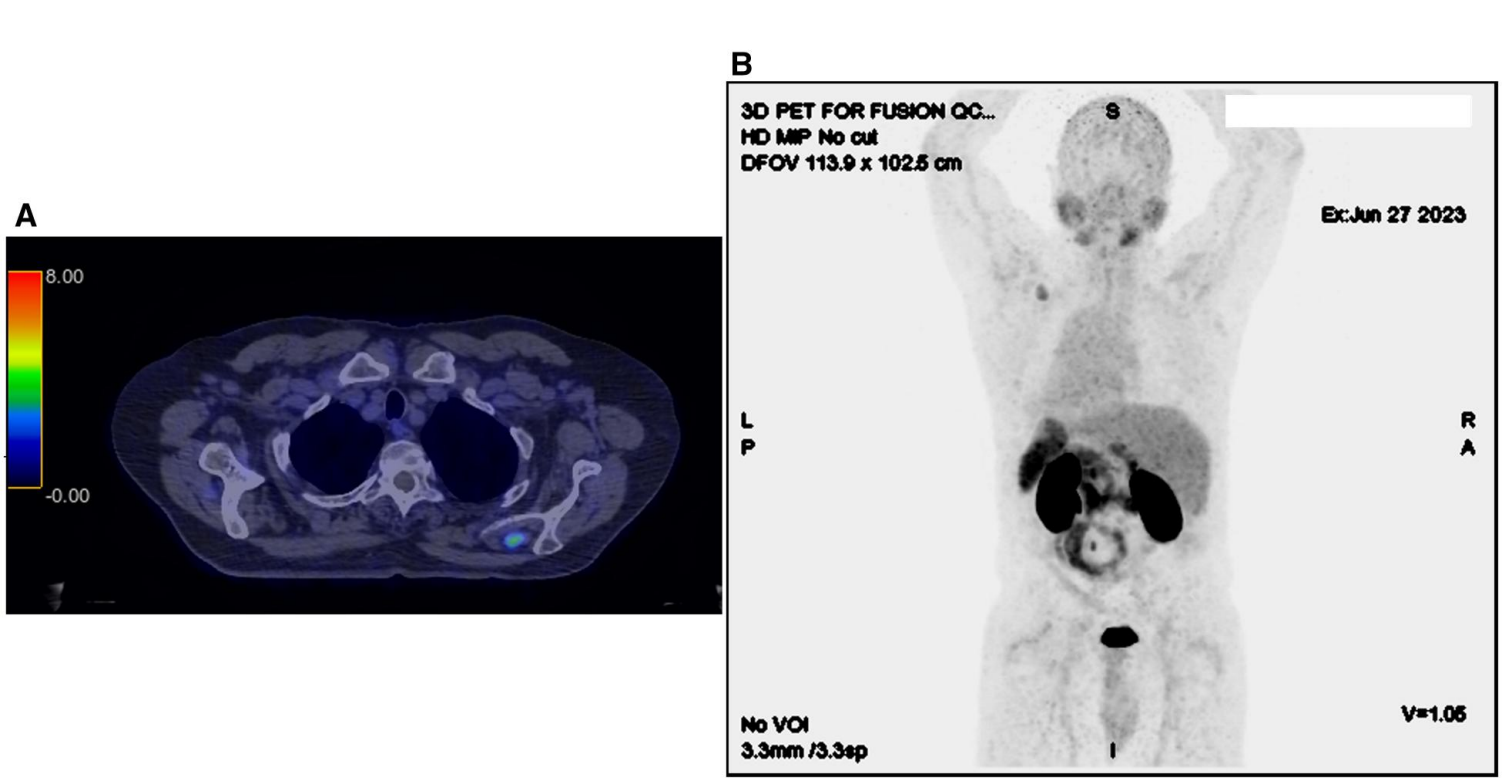
Whole body PSMA-PET/CT. (A) Moderate PSMA-PET avidity in the left supraspinatus. (B) No other PSMA-avid lesions.

We present a case of a 60-year-old male with a prostate-specific membrane antigen PET (PSMA-PET) avid left supraspinatus mass, which was incidentally detected on staging PSMA-PET performed for a localized intermediate risk prostate cancer (T1cN0M0 Gleason 3 + 4 = 7 initial PSA 5.9 µg/L), with a maximum SUV 4.3 and measured 20 × 12 × 23 mm on the co-registered CT. No PSMA-avid lesion was seen in the prostate or elsewhere (Figure 1). The case was reviewed at the local urology multidisciplinary meeting which included radiology review by a dual-trained nuclear medicine physician and radiologist with an interest in PSMA-PET. The consensus recommendation was for further ultrasound and MRI to characterise the lesion, image-guided biopsy, and discussion at a tertiary sarcoma multidisciplinary meeting.

**Figure 2.**
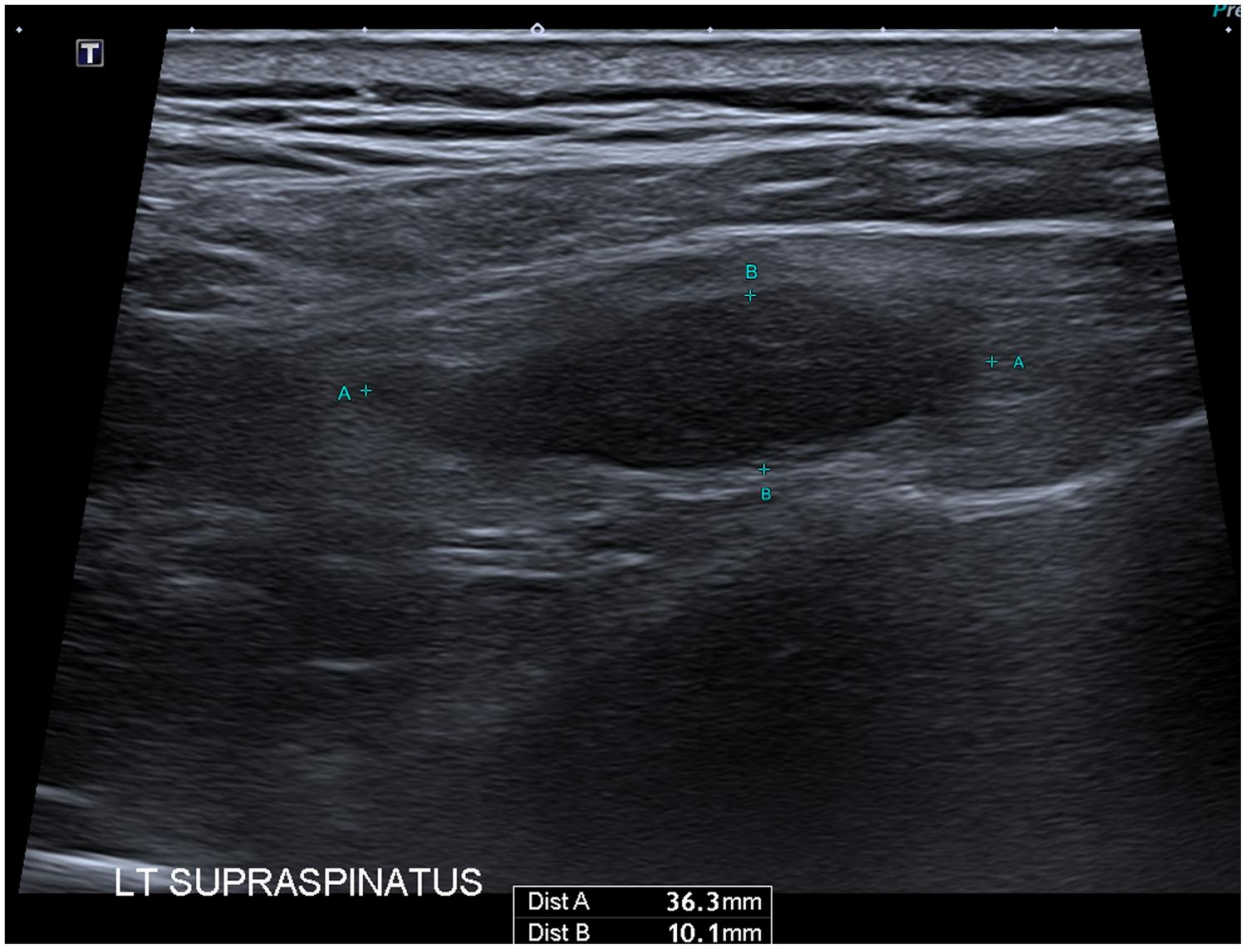
Anechoic on ultrasound.

**Figure 3.**
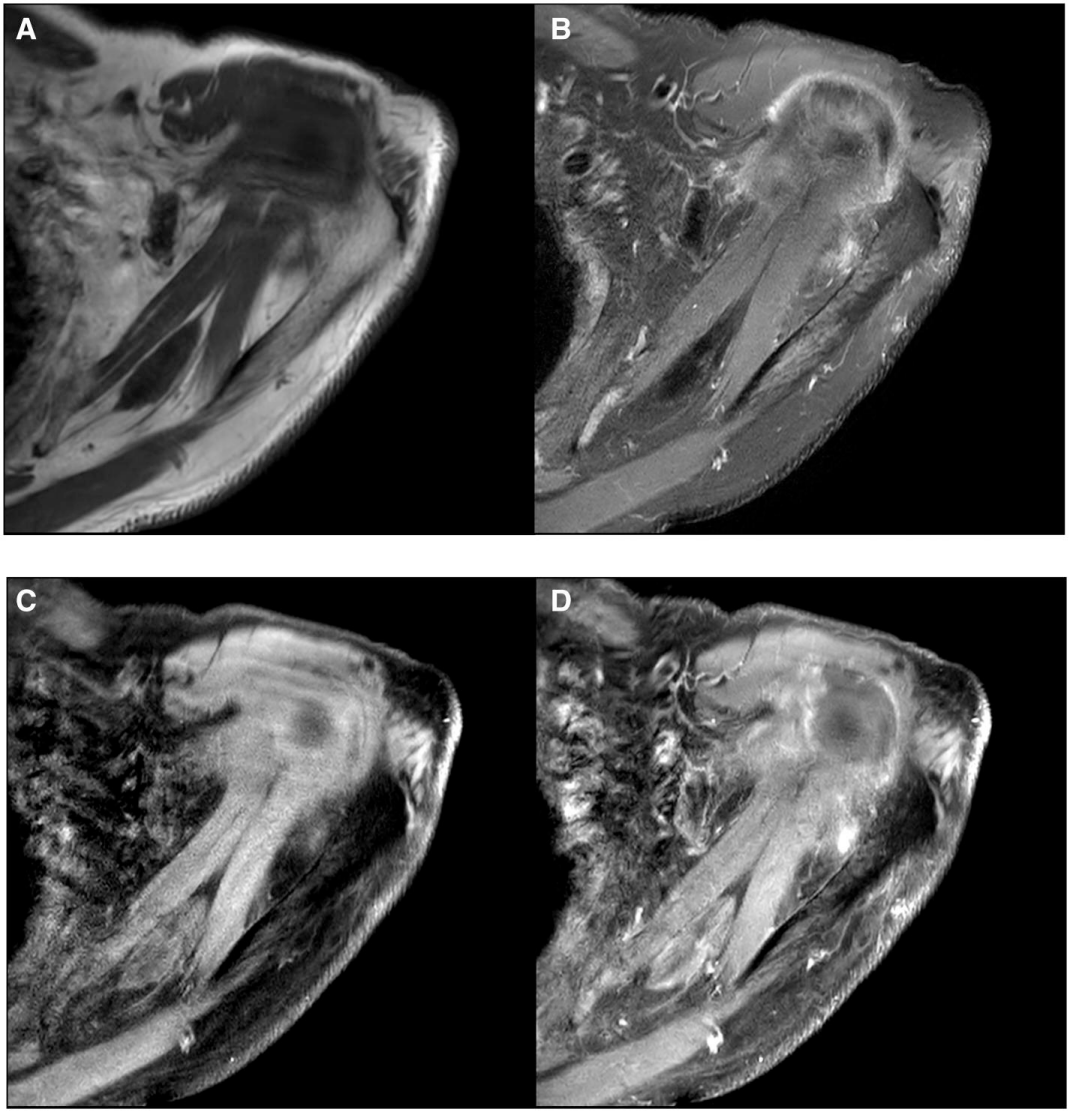
MRI left shoulder. (A) Axial T1: iso to hypointense to muscle. (B) Axial T2 fat-saturated: hypointense to muscle. (C) Axial T1 fat saturated: iso to hypointense to muscle. (D) Axial T1+contrast: enhancement with gadolinium.

**Figure 4.**
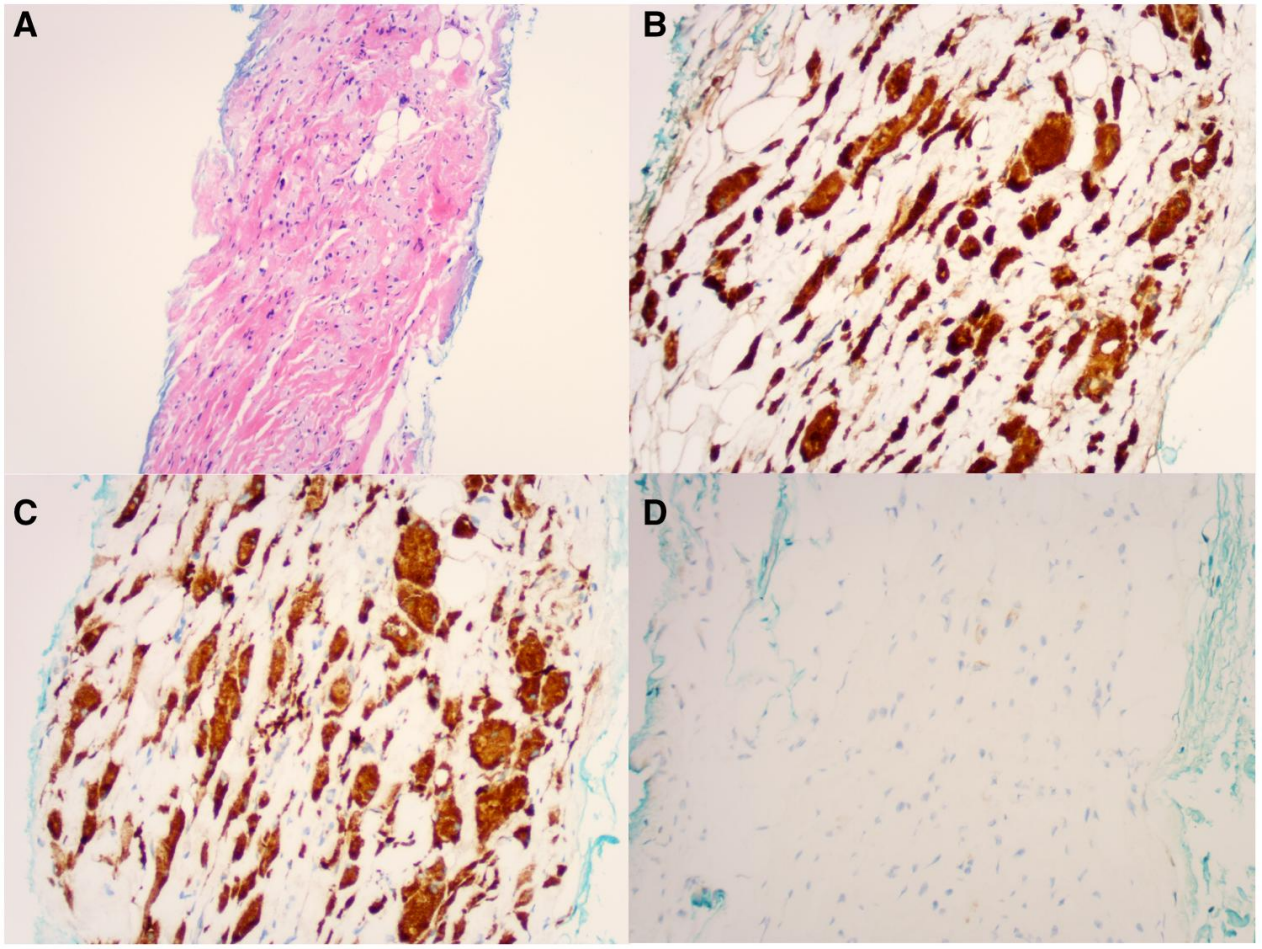
Histopathology and immunohistochemical staining (IHC) of core biopsy. (A) Haematoxylin and Eosin. (B) S100 IHC positive. (C) CD68 IHC positive. (D) Pan-cytokeratin IHC negative.

Targeted ultrasound of the left shoulder identified a 36 × 10 × 18 mm anechoic lesion with low internal vascularity, and was reported as indeterminate in nature (Figure 2). MRI of the left shoulder showed a 35 × 12 × 15 mm, ovoid, T1 hypo-intense, T2 hypo-intense lesion in the supraspinatus belly, with mild gadolinium enhancement (Figure 3). Ultrasound-guided core biopsy and histopathological analysis showed cells with abundant cytoplasm and cytoplasmic granularity, with positive immunoperoxidase staining for CD68 and S100, negative staining for pan cytokeratins: favoured to represent granular cell tumour (Figure 4). The case was reviewed at a tertiary sarcoma multidisciplinary meeting. The histopathology was confirmed as a benign granular cell tumour by a dedicated sarcoma pathologist. The patient was offered either complete excision, or repeat interval surveillance imaging in 6 months. The patient has requested excision, and is pending surgical consultation. He is also currently undergoing definitive radiation therapy to his prostate cancer.

## Discussion

To our knowledge, there are no published reports of PSMA-PET avidity in a granular cell tumour. We present the first case report of PSMA-PET avidity in a granular cell tumour. Granular cell tumour is a rare, mostly benign, soft tissue tumour with neuroectodermal differentiation, also known as an abrikossoff tumour or granular cell myoblastoma.^1^

It has a slight female predilection, a mean age at diagnosis at 45 years old, and is located predominantly in skin, peripheral soft tissues and alimentary tract (66% oesophagus), with rarer presentations in the breast, vulva, larynx and bronchus.^2^

Histologically it is composed of plump cells with eosinophilic granular cytoplasm and is graded according to either the Fanburg-Smith or Nasser criteria.^1^^,^^3^ Approximately 93% of the reported cases are benign, 7% have uncertain malignant potential on histopathology and only 2.5% of all granular cell tumours are documented to have metastasized.^2^

On ultrasound, it is usually hypoechoic, and may variably display posterior acoustic shadowing, posterior enhancement and internal hypervascularity.^4^ It is usually isodense to muscle on CT^5^ and non-glucose avid on FDG-PET, although malignant granular cell tumours have been reported to be strongly glucose-avid.^6^ On MRI it is usually T1 isointense, T2 iso or hypointense, and enhances with gadolinium contrast.^7^

PSMA-PET is increasingly used in staging, surveillance and treatment planning for prostate cancer as it can result in a change to management versus conventional imaging up to 50% of the time.^8^ PSMA-PET scans have been reported to demonstrate significant uptake in benign and malignant entities apart from prostate cancer.^9^ This case demonstrates the utility of obtaining histological confirmation of PSMA-PET avid lesions found in unusual locations for prostate cancer metastases. Prostate cancer typically metastasizes to abdomino-pelvic lymph nodes and bone, but rarely to other sites such as muscle, skin and peripheral soft tissues.^10^ In this case, if not for histologic confirmation, the patient may have been classified as stage IV in the presence of a presumed PSMA-avid distant metastasis, and thus denied curative intent therapy to an otherwise localized prostate cancer, and also be subject to highly toxic life-long androgen deprivation therapy as the standard of care for metastatic prostate cancer. Additionally, with the increasing use of oligometastatic-directed stereotactic ablative radiotherapy in the oligometastatic setting, histologic confirmation is not always obtained prior to oligometastatic-directed ablative therapy. In this case, an unnecessary and potentially toxic treatment would have been avoided if a biopsy was obtained. By performing image-guided core biopsy, we were able to exclude metastatic involvement, confirm localised disease, and offer curative intent management.

## Learning points

1. This case reminds the clinician that not all PSMA-PET avid lesions are malignant.
2. Multidisciplinary input including a nuclear medicine physician with interest in PSMA-PET is crucial to provide guidance on the possible benign and malignant entities which are PSMA-PET avid.
3. Obtaining histopathologic confirmation is especially recommended when the site of PSMA-avidity is in an unusual location for prostate cancer metastasis.
4. Granular cell tumours are a mostly benign, but rarely malignant entity which may be PSMA-avid.

## Conclusion

To our knowledge, this is the first case report of PSMA-PET avidity in a granular cell tumour. We add to the growing literature documenting PSMA-PET avidity in benign and malignant lesions apart from prostate cancer.
